# The efficacy of rhBMP-2 loaded hydrogel composite on bone formation around dental implants in mandible bone defects of minipigs

**DOI:** 10.1186/s40824-020-0183-9

**Published:** 2020-02-03

**Authors:** Hao-Zhen Lyu, Jae Hyup Lee

**Affiliations:** 1 Department of Orthopedic Surgery, College of Medicine, SMG-SNU Boramae Medical Center, Seoul National University, Boramae-ro 5-gil 20, Dongjak-gu, Seoul, 07061 Korea; 2 Institute of Medical and Biological Engineering, Medical Research Center, Seoul National University, Seoul, Korea

**Keywords:** Bone morphogenetic protein-2, Hydrogel, Minipig, Osteogenesis, Osseointegration

## Abstract

**Background:**

In dental or orthopedic surgery, bone substitutes are inserted with implants to promote osteogenesis and enhance osseointegration. The purpose of this research was to evaluate the efficacy of rhBMP-2 (recombinant human bone morphogenetic protein-2) loaded hydrogel composite for bone formation around dental implant in minipig mandible bone defect models.

**Methods:**

We made bone defects with a diameter of 4 mm in minipig mandibles and inserted implants of the same size, to mimic the cases of inserting the screws in the bone defect or poor-quality bone. The rhBMP-2 (300 μg) loaded hydrogel composite (0.5 cc) inserted in the bone defect with the implant in the rhBMP-2 group. After 4 weeks, the mandibles were harvested to evaluate the new bone mass around implants using plain radiographs, micro-CT, and histology.

**Results:**

The micro-CT analysis result showed that the quantity of new bone generation around the implant in the rhBMP-2 group was greater than that in the other groups. Comparing the ratios of bone to implant area in three groups by histology, the amount of newly formed bone in the rhBMP-2 group was the most.

**Conclusion:**

The rhBMP-2 loaded hydrogel composite promotes osteogenesis around dental implant in minipig mandible bone defect, and enhance osseointegration between the dental implant and host bone.

## Background

In dental and orthopedic surgery, when a patient has traumatic bone defects, osteoporosis, or bone resorption and needs an internal fixation implant, increasing the stability of the implant and the subsequent bone fusion rate is pivotal. Autogenous bone and bone substitutes are most commonly used to repair bone defects or to increase bone fusion rates. Choices of bone substitutes include demineralized bone matrix, hydroxyapatite, calcium phosphate, tricalcium phosphate, and calcium sulphate [1–3]. However, these substitutes all lack osteoinductivity.

Bone morphogenetic proteins (BMPs), members of the transforming growth factor-β (TGF–β) superfamily, received extensive attention after being demonstrated to have the ability to stimulate bone formation [4–7]. And BMPs are added to synthetic bone in order to improve the osteoinductivity of bone substitutes. In recent years, rhBMPs has been produced using recombinant technology. It has been demonstrated in animal model and clinical application that local use of rhBMP-2 can also stimulate bone formation [8, 9]. The effect of rhBMP-2 on osteogenesis is related to the duration of release [10]. As a soluble protein, if rhBMP-2 is directly placed into mice, rhBMP-2 would be retained for 3 days [11]. Although rhBMP-2 used alone also can play a role in bone generation, its persistence time is too short for bone healing. On the contrary, the rhBMP-2 incorporating gelatin hydrogels could release rhBMP-2 for more than 30 days [11]. Thus, the carrier is necessary to extend the release time of rhBMP-2. In clinical cases, most bone defects are irregular in shape. An injectable carrier, such as a gel, be remodeled according to the shape of the defect and make good adhesion to the host bone and implant surface. Therefore, the injectable carrier is more suitable for irregular bone defects.

As the main component of the extracellular matrix (ECM), hyaluronic acid (HA) is a naturally-derived and injectable carrier of rhBMP-2. However, because of its diffusive physical properties, HA releases rhBMP-2 too rapidly. The butanediol diglycidyl ether (BDDE) as a crosslinking agent is thus used to improve the internal stability and physical appearance of HA [12–14]. The porous tricalcium phosphate (TCP) microspheres exhibit osteoconduction, osteointegration, and bioactivity for binding to rhBMP-2. So, it can be used as bone substitutes and rhBMP-2 carriers [15].

If the surgical area has a bone defect in the clinic, we need to implant the bone grafts into the bone defect first, and surgical treatment should be carried out after the bone defect has healed. However, when surgery must be performed on the bone defect or when a bone defect occurs during surgery, the bone substitutes could be placed with the implant to promote bone formation. Few studies have been conducted assessing the osteogenesis effect of BMP-2 in the bone defect or poor-quality bone.

Due to the anatomical and bone biological similarities with humans, minipigs have been extensively used for the evaluation of bone defect healing and bone regeneration [16]. In addition, the bone regeneration rate in minipig mandible is comparable to that in humans [16, 17]. Therefore, we made bone defects in the minipig mandible and placed the screws with rhBMP-2 to mimic the case that inserting the implant into the bone defect in the clinic. The purpose of this study was to evaluate the efficacy of rhBMP-2 loaded hydrogel composite on osteogenesis around the implant in the bone defect.

## Methods

### Preparation of rhBMP-2

Escherichia coli-derived rhBMP-2 was supplied from Daewoong Pharmaceutical (Seoul, Korea). The rhBMP-2 is a disulfide-linked dimeric-protein molecule with 115 amino acids and provided as a lyophilized white powder containing glutamic acid, glycine, sucurose, polysorbate 80, sodium chloride and sodium hydroxide. It was dissolved in water for injection just before use. The rhBMP-2 solution has a pH 4.5 and is clear, colorless and essentially free form plainly visible particulate matter.

### Preparation of HA powder gel and β-TCP microspheres composite

Cross-linked hyaluronate base powder gel and resorbable β–tricalcium phosphate (Cerectron Co., Korea; β-TCP) were used together as an injectable carrier of rhBMP-2. The powder gel was prepared by cross-linking sodium hyaluronate ([C_14_H_20_NO_11_Na]n, Bioland Co., Korea) of three million Daltons (Da) with butanediol diglycidyl ether (C_10_H_18_O_4_, Sigma-Aldrich, USA; BDDE), a cross-linking agent under the condition of HA 2.7 wt% and BDDE 10% [18, 19]. After cross-linking, the remaining BDDE was removed by dialysis with 1X phosphate buffered saline (Sigma-Aldrich, USA; PBS) for 5 days. After dialyzing, the HA hydrogel was undergone lyophilization, crushing and sorting to make it as the powder gel with a size of 500 μm or less. The β-TCP beads were prepared by spray-drying to spherical particle forms and then sintered at a high temperature of 1250 °C and then sieved at 45 to 75 μm. The HA based powder gel and β-TCP beads were uniformly mixed at a ratio of 9:1 and filled in a syringe [9, 15].

### rhBMP-2 loaded hydrogel composite

A prefilled syringe containing hydrogel and another syringe containing rhBMP-2 solution was connected with a 2-way connector and mixed immediately prior to application into an animal model [15]. The amount of hydrogel composite injected per defect was 0.05 cc including 300 μg of rhBMP-2.

### In vivo animals

Five 18–20 months old Yucatan male minipigs (35–40 kg) were used in this research. The specific pathogen-free (SPF) minipigs were supplied by Medi-Kinetics Company Ltd. (Pyeongtaek, Korea). This research was approved by the Standing Ethical Committee for Animal Research of the Laboratory in the Clinical Research Institute of Medi-Kinetics (IACUC NO. 110525–001). All animals had a period of more than a week for acclimation in standard cages. Unnecessary stress and discomfort were avoided during the research. The animals were exposed to a 12-h light/dark cycle, 20 ± 10 °C temperature, and 40 ± 10% humidity.

### Mandible defect models and implantations

A minipig was administered general anesthetized via endotracheal intubation. The molars and premolars were extracted on both sides of the mandible. And the soft tissues around the wound were sutured in order to cover the defect holes. The minipig was then intravenously injected with 1 g cefazolin. The remaining four minipigs were anesthetized and operated in the same method. Following the surgery, the minipigs were raised in standard cages for 4 weeks. The minipigs fasted for 3 days after operation, were fed liquid food in the next 7 days and were given a soft diet thereafter until the second surgery.

The five minipigs were anesthetized in the same manner as in the first operation, and the covered soft tissue was incised to expose the previous extraction defects. Afterwards, consistent bone defect models with a diameter of 4 mm were drilled at the tooth extraction sites on both sides of the mandible. There were 20 bone defect models divided randomly into three groups. The dental implants (provided by MegaGen, 4 mm diameter × 8.5 mm length, Seoul, Korea) were inserted as follows:dental implants only (six implants, the implant group), dental implants with hydrogel (eight implants, the hydrogel group), or dental implants with rhBMP-2 (300 μg) loaded hydrogel composite (six implants, the rhBMP-2 group). The implants were completely embedded into the mandible defects and the surrounding soft tissue was sutured in order to cover the implants. Animals were intravenously injected with 1 g cefazolin. After implantations, the minipigs were raised freely in cages for 4 weeks. All animals fasted for the first 3 days, then were fed liquid food for the next 7 days and were given a soft diet until they were euthanized.

### Plain radiographs evaluation

The mandibles of minipigs were harvested after euthanasia. The plain radiographs were obtained at 45 kV for 12 ms. The loosening and pullout of implants and bone quality around implants were observed.

### Micro-CT evaluation

Micro-CT (Skyscan 1173, Bruker, Kontich, Belgium) was performed on the complete mandibles including implants at 130 kV, 30 μA, at a medium resolution of 40 μm, with a brass filter. We selected the threads in the middle of the screw and the region of the screw grooves as the ROI (region of interest). The parameters of newly formed bone quantities in ROI were obtained using a CT analysis system, such as percent bone volume (BV/TV), trabecular number (Tb.N), trabecular thickness (Tb.Th), specific surface (BS/BV), trabecular bone pattern factor (Tb.pf) and trabecular separation (Tb.Sp).

### Histological evaluation

The mandible specimens containing implants were fixed with formalin for 5 days and every specimen was divided into two parts. The gross specimens after trimming were washed for 6 h in a cassette and dehydrated in 100% alcohol. We then put them into methacrylate-based chemical curing resin and stirred for 2 days. Next, the specimens were added, stirred and embedded by dissolving in the benzoyl peroxide. The blocks were trimmed again and cut using an EXAKT cutting instrument (BS-3000 N) at 4-μm thickness along the sagittal plane of the implant. The specimens were then ground with an EXAKT 4110 grinding machine, and the specimen was attached to an acrylic slide and underwent hematoxylin and eosin (H&E) staining. The new bone generation between the fixture and original mandible bone was subsequently observed. The bone-to-implant area ratios and the bone-to-implant contact ratios were measured to evaluate osteointegration by light microscope.

### Statistical analysis

One-way ANOVA and Bonferroni’s post-hoc tests (SPSS version 23; IBM) were performed for the normally-distributed data in this research. A *P*-value < 0.05 was considered to be statistically significant.

## Results

### Gross findings

No death, infection, or tissue necrosis occurred in the experiment animals. Minipigs were not given the protective measures of the implant that conventionally provided for the patient after surgery in the dental clinic. Twelve implant failures (dropped out or broken off) occurred during the postoperative observation period. In the implant group and the hydrogel group, we saw newly formed osteoid tissue merely filling the defect space. In the rhBMP-2 group, we observed new osteoid tissue filling the defect space and covering the implants.

### Plain radiographs results

**Fig. 1:**
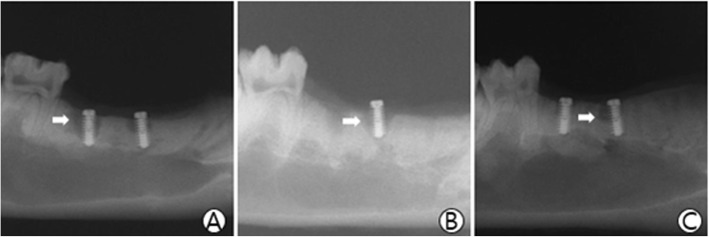
Radiograph finding. The white arrows indicate the radiolucent shading area around the implants. **a** The implant group; **b** The hydrogel group; **c** The rhBMP-2 group

The host bone damages and the bone quality around the implants were observed by the radiolucent shading. We saw the radiolucent shading area in all three groups (Fig. 1).

### Micro-CT results

**Fig. 2:**
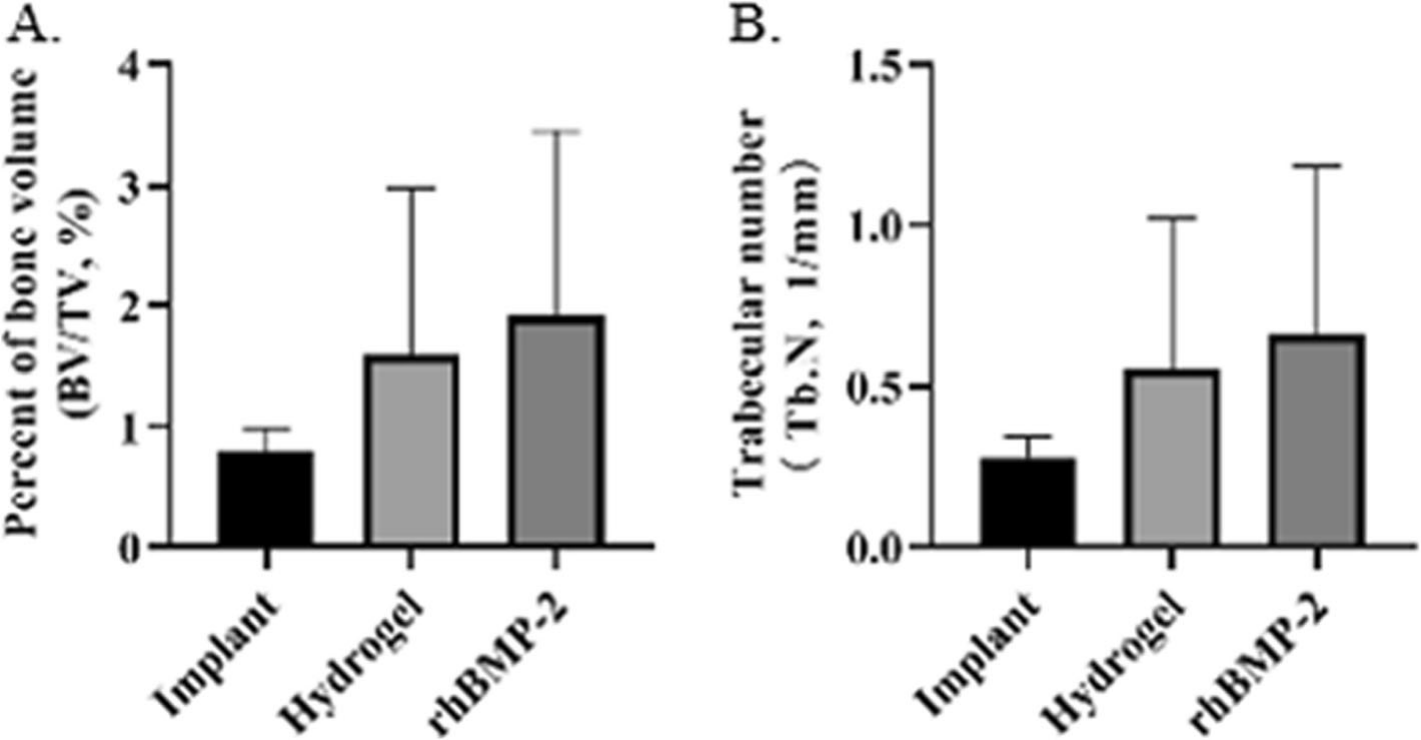
Percent of bone volume (BV/TV) and trabecular number (Tb.N) by micro-CT analysis. **a** BV/TV: implant vs rhBMP-2, *P* = 0.055; **b** Tb.N: implant vs rhBMP-2, *P* = 0.055

The micro-CT results were used for the quantitative assessment of osteogenesis. The percentage of bone volume in the rhBMP-2 group (1.91 ± 1.54), was the highest, followed by the hydrogel group (1.59 ± 1.38, *P* = 1.0), and the implant group (0.8 ± 0.19, *P* = 0.055) was the lowest (Fig. 2a). The trabecular number in the rhBMP-2 group was higher (0.66 ± 0.52) than that in the other groups (the hydrogel group, 0.55 ± 0.47, *P* = 1.0; the implant group, 0.28 ± 0.07, *P* = 0.055) (Fig. 2b). The other results of micro-CT analysis are listed in Table 1.

**Table 1:** Micro-CT results for osteogenesis around implants

Group(*n* = 14)	BV/TV	BS/BV	Tb.N	Tb.Th	Tb.Sp	Tb.Pf
Implant group	0.80 ± 0.19	315.09 ± 21.10	0.28 ± 0.07	0.03 ± 0.00	0.34 ± 0.04	70.76 ± 8.04
Hydrogel group	1.59 ± 1.38	288.27 ± 54.68	0.55 ± 0.47	0.03 ± 0.00	0.31 ± 0.06	56.71 ± 21.30
rhBMP-2 group	1.91 ± 1.54	286.31 ± 42.49	0.66 ± 0.52	0.03 ± 0.00	0.29 ± 0.07	54.03 ± 18.91
*P*-value	0.055	0.228	0.055	0.599	0.106	0.04

Values are presented as mean ± standard deviation

*P*-value: the rhBMP-2 group vs the implant group

*BV/TV (%)* percent bone volume (bone volume/total volume), *BS/BV (1/mm)* bone surface/bone volume, *Tb.Pf (1/mm)* trabecular bone pattern factor, *Tb.Th (mm)* trabecular thickness, *Tb.N (1/mm)* trabecular number, *Tb.Sp (mm)* trabecular separation

### Histological results

We observed osteogenesis in the implant groove on undecalcified histological specimens. The bone-to-implant area ratios in the three groups were 13.92% (the rhBMP-2 group, Std.12.34), 2.42% (the hydrogel group, Std.7.51), and 6.96% (the implant group, Std.13.43), respectively. The area ratio in the rhBMP-2 group was the highest, and there was a statistically significant difference when compared to the ratio in the hydrogel group (*P* = 0.002). Moreover, the bone-to-implant contact ratios in the rhBMP-2 group (3.40 ± 4.27%) were higher than that in the hydrogel group (1.19 ± 5.83%, *P* = 1.0) or the implant group (2.92 ± 8.59%, *P* = 0.724). The results are listed in Table 2.

**Table 2:** Histological results for osteointegration

Group (*n* = 24)	Bone to implant contact ratio	Bone to implant area ratio
Implant group	2.92 ± 8.59	6.96 ± 13.43
Hydrogel group	1.19 ± 5.83	2.42 ± 7.51
rhBMP-2 group	3.40 ± 4.27	13.92 ± 12.34^*^

Values are presented as mean ± standard deviation

^*^Significantly greater than the hydrogel group (*P* < 0.05)

## Discussion

Due to its excellent osteoinductivity, rhBMP-2 is used in spinal fusion, repairing a long bone defect, dental and maxillofacial surgery. Some researchers have pointed out that the sustained release of rhBMP-2 is necessary for bone formation, but that the initial burst release of rhBMP-2 is a crucial step for the entire bone formation process [20, 21]. Therefore, the osteogenic efficiency of rhBMP-2 is related to the release mechanism.

In this study, the rhBMP-2 loaded porous β-TCP microsphere-hyaluronate base powder gel composite was inserted into mandible defects with dental implants. When the composite was placed into the defect, the rhBMP-2 in hydrogel was released to the contact interface with mandible bone by diffusion, then the initial burst release occurred. Our previous research reported that the rhBMP-2 in porous β-TCP was slowly released to the hyaluronate base powder gel at first, and that the rhBMP-2 was then released from powder gel to the surrounding tissues [15, 22]. Therefore, the rhBMP-2 loaded hydrogel composite used in this study could release rhBMP-2 slowly as the composite was absorbed.

**Fig. 3:**
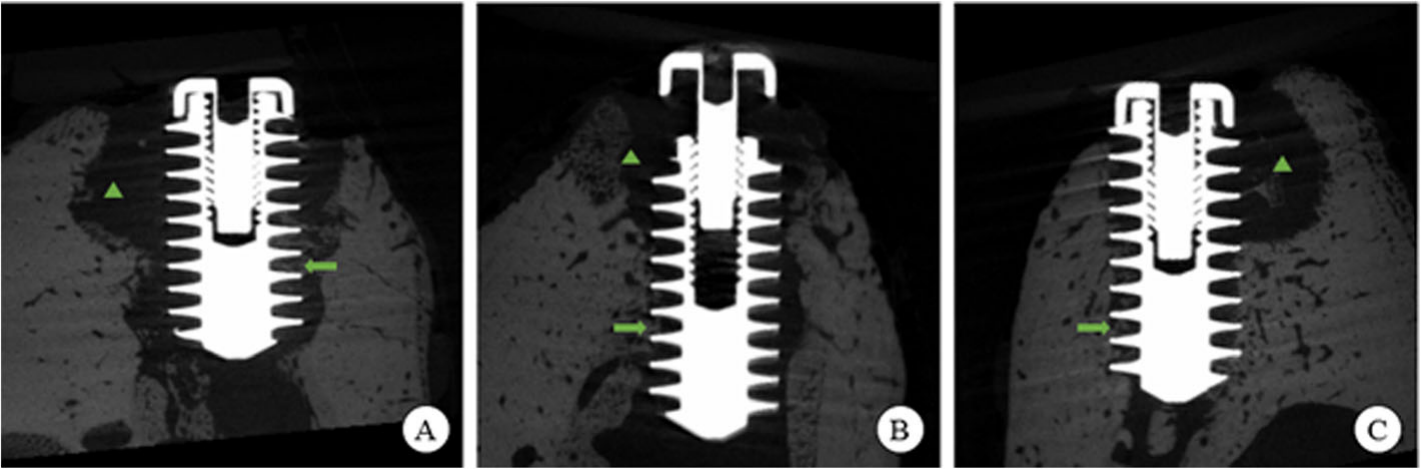
Micro-CT finding. The newly formed bone in the rhBMP-2 group is more than that in the other groups. The green arrows indicate new bone that growth into the implant threads. The green triangles indicate the damages of host bone. **a** The implant group; **b** The hydrogel group; **c** The rhBMP-2 group

In this research, we made 4 mm diameter defects in the minipig mandible and inserted implants of the same diameter size, in order to imitate the situation of inserting implant into the bone defect or poor-quality bone. There was little bone tissue around the implant threads. Due to the weak stability of implants, the minipigs caused damage to the host bone in the periphery of defects by the friction between implant and bone (Fig. 3). The implants dropped out or were broken off when the minipigs were biting cages and feeding. The micro-CT analysis showed that the quantity of new bone generation around the implant in the rhBMP-2 group was greater than that in the other groups. These results indicated that the rhBMP-2 loaded hydrogel composite can promote bone growth in the poor-quality bone or under the poor initial stability of the implant.

**Fig. 4:**
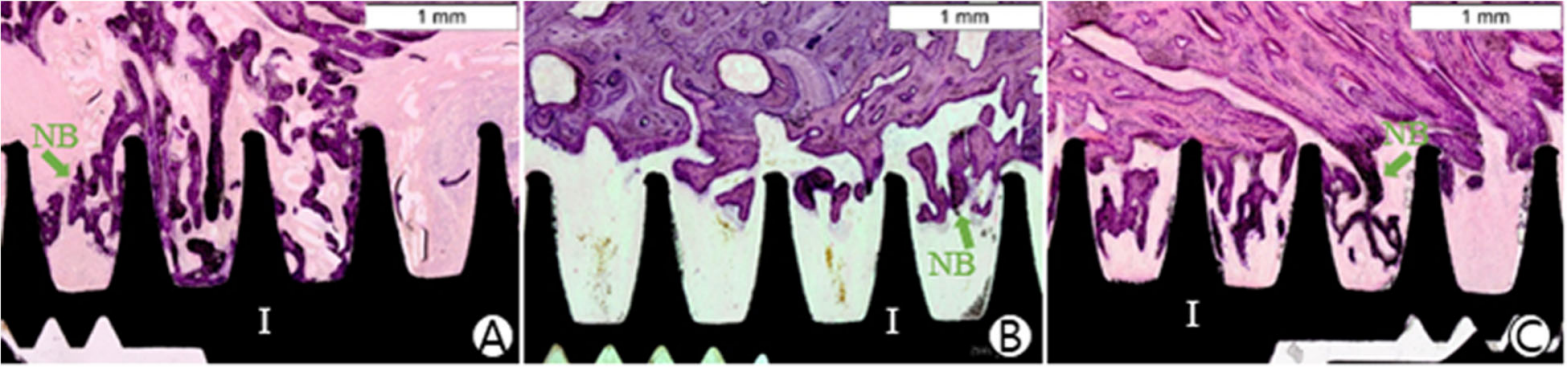
Histological finding. The implant osseointegration in the rhBMP-2 group was the best. Compared to the implant group, the osseointegration of the implant in the hydrogel group was the worst. The green arrows indicate new formed bone around the implant. Hematoxylin and eosin stain(× 40). Scale bar = 1 mm. I: implant; NB: new bone. **a** The implant group; **b** The hydrogel group; **c** The rhBMP-2 group

The stability of the implant is related to the integration between the implant and the bone. The bone-to-implant contact ratio provided by the histological analysis is considered as an indirect method for evaluating osteointegration. The bone-to-implant contact ratio in the rhBMP-2 group was the highest, followed by the implant group, and the hydrogel group was the least (Table 2 and Fig. 4). Comparing the bone-to-implant area ratios in the implant grooves, the amount of newly formed bone tissue in the rhBMP-2 group was the most among the three groups (Fig. 4). These results confirmed that rhBMP-2 loaded hydrogel composite could stimulate osteogenesis and enhance osteointegration of the implant.

It is noteworthy that the bone-to-implant contact ratio in the hydrogel group was less than that in the implant group, even though this trend was not statistically significant. With the release of rhBMP-2 and the absorption of carrier, a new bone formation progressed from the interface between the mandible host bone and carrier in the direction of the implant. This phenomenon demonstrates that if the absorption of the carrier is slower than the formation of new bone, the residual carrier will stay around the implant thread and impede bone cell ingrowth. Therefore, the absorption rate of the carrier cannot be slower than the generation rate of new bone.

A limitation of this research is the small sample size, so some results are biased. The other limitation is the weak initial stability of the implant, which makes a negative effect on new bone formation. Therefore, the results obtained in this harsh environment are more conducive to extrapolating the clinical efficacy of rhBMP-2 in the bone defect.

## Conclusion

The rhBMP-2 loaded hydrogel composite can promote new bone formation in the mandible bone defect, and enhance osseointegration between the dental implant and host bone.

## Data Availability

All data generated or analyzed in this study are included in this published article.

## Abbreviations

rhBMP-2: Recombinant human bone morphogenetic protein-2 HA Hyaluronic acid
TCP: Tricalcium phosphate
